# Supplementary material for: Novel Driver Strength Index highlights important cancer genes in TCGA PanCanAtlas patients
Source: PeerJ. 2022 Aug 11;10:e13860. doi: 10.7717/peerj.13860 (PMC9375969; doi:10.7717/peerj.13860)
Supplement: Supplemental Information 2 [file peerj-10-13860-s002.zip › COHORTS/genes plots/detailed/2021_8_16_14_9_distribution_events_detailed_males_41.pdf]

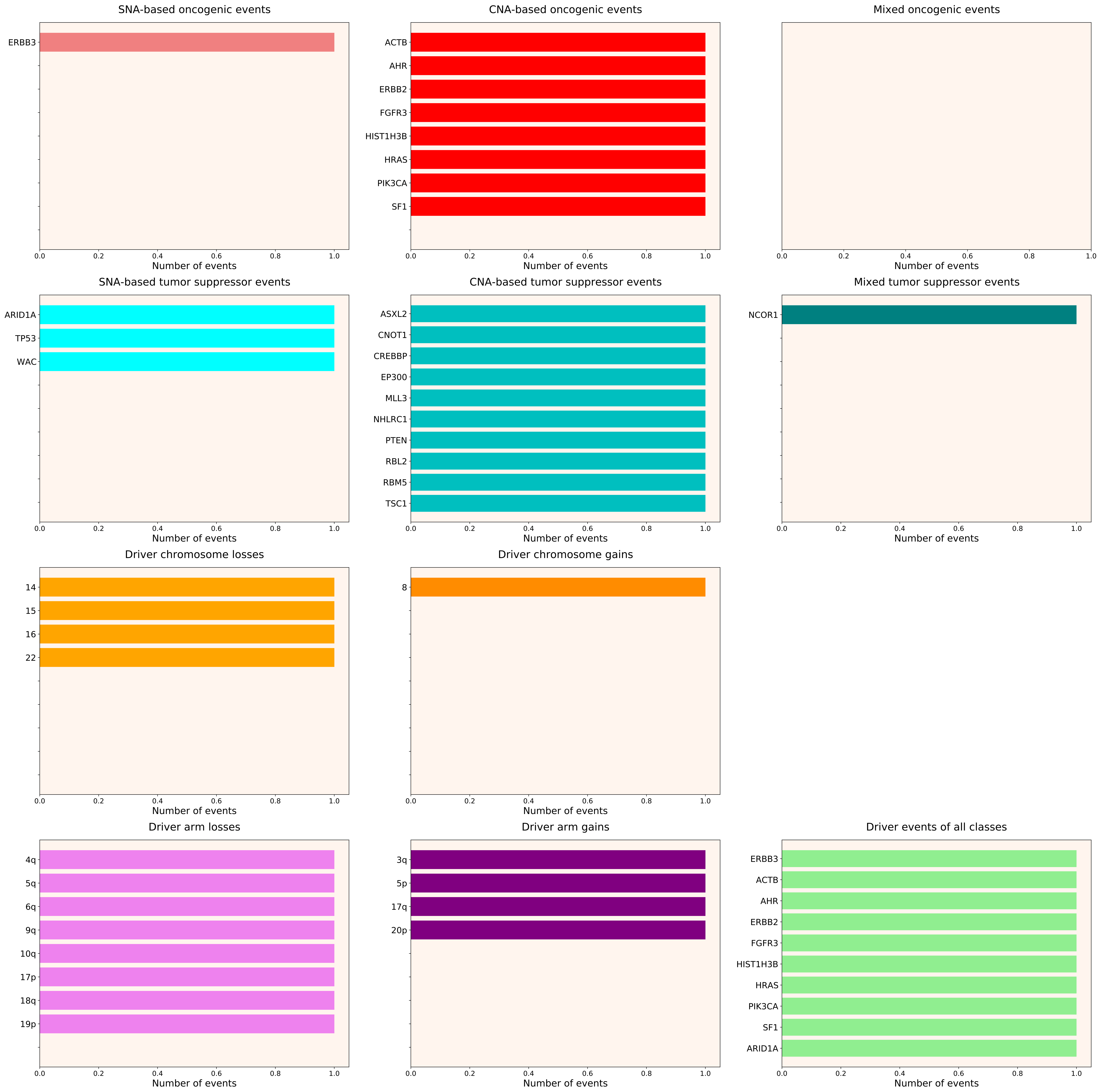
SNA-based oncogenic events
ERBB3
CNA-based oncogenic events
ACTB
AHR
ERBB2
FGFR3
HIST1H3B
HRAS
PIK3CA
SF1
Mixed oncogenic events
SNA-based tumor suppressor events
ARID1A
TP53
WAC
CNA-based tumor suppressor events
ASXL2
CNOT1
CREBBP
EP300
MLL3
NHLRC1
PTEN
RBL2
RBM5
TSC1
Mixed tumor suppressor events
NCOR1
Driver chromosome losses
14
15
16
22
Driver chromosome gains
8
Driver arm losses
4q
5q
6q
9q
10q
17p
18q
19p
Driver arm gains
3q
5p
17q
20p
Driver events of all classes
ERBB3
ACTB
AHR
ERBB2
FGFR3
HIST1H3B
HRAS
PIK3CA
SF1
ARID1A
Number of events
0.0
0.2
0.4
0.6
0.8
1.0
